# Supplementary material for: Burden, risk factors, and forecasts of gout in BRICS countries, 1990–2021: Insights from the Global Burden of Disease Study 2021
Source: PLoS One. 2025 Sep 24;20(9):e0332265. doi: 10.1371/journal.pone.0332265 (PMC12459839; doi:10.1371/journal.pone.0332265)
Supplement: Attachment — Submitted filename: Response to Reviewers.docx [file pone.0332265.s002.docx]

**Response to the Editor and Reviewers**

Dear Dr.Yang and reviewers,

We sincerely appreciate your time and the opportunity to revise and improve our manuscript entitled “Burden, risk factors, and forecasts of gout in BRICS countries, 1990–2021: insights from the Global Burden of Disease Study 2021” (Manuscript ID: PONE-D-25-27682). Thank you for coordinating the review process and for your constructive suggestions. We have carefully considered all comments and revised the manuscript accordingly. Below, we provide a detailed response to each of the reviewers’ suggestions and concerns.

We hope the revised version of our manuscript addresses the concerns raised and meets the journal’s standards. All changes have been highlighted in the revised manuscript for ease of reference.

**Journal Requirements:**

When submitting your revision, we need you to address these additional requirements.

1. Please ensure that your manuscript meets PLOS ONE's style requirements, including those for file naming. The PLOS ONE style templates can be found at

<https://journals.plos.org/plosone/s/file?id=wjVg/PLOSOne_formatting_sample_main_body.pdf> and [https://journals.plos.org/plosone/s/file?id=ba62/PLOSOne_formatting_sample_title_authors_affiliations.pdf](https://track.editorialmanager.com/CL0/https:%2F%2Fjournals.plos.org%2Fplosone%2Fs%2Ffile%3Fid=ba62%2FPLOSOne_formatting_sample_title_authors_affiliations.pdf/1/010f01980824da59-dd13a9fc-501d-4cbe-af48-c7620cb327da-000000/cjQYdkc2KzboB4nhoyenDee4262dEti372w-ac-JaAk=219).

**Reply 1:** We have formatted the manuscript according to the journal’s style guidelines and ensured correct file naming for all submitted documents, including: Response to Reviewers; Revised Manuscript with Track Changes; Manuscript (clean version).

2. We note that the grant information you provided in the ‘Funding Information’ and ‘Financial Disclosure’ sections do not match.

When you resubmit, please ensure that you provide the correct grant numbers for the awards you received for your study in the ‘Funding Information’ section.

**Reply 2:** We have updated the “Funding Information” section to ensure consistency with the “Financial Disclosure.” The correct grant number has been provided, and the funding statement now reads: “This study was supported by Guangxi Clinical Research Center for Urology and Nephrology (grant No. Guike20297081)”.

3.  Thank you for stating the following financial disclosure:

 [This study was supported by Guangxi Clinical Research Center for Urology and Nephrology (grant No. Guike20297081).].

Please state what role the funders took in the study.  If the funders had no role, please state: ""The funders had no role in study design, data collection and analysis, decision to publish, or preparation of the manuscript.""

If this statement is not correct you must amend it as needed.

Please include this amended Role of Funder statement in your cover letter; we will change the online submission form on your behalf.

**Reply 3:** The corresponding author (Jiwen Cheng), who is the principal investigator of the grant, was involved in the study design, decision to publish, supervision and manuscript preparation.

4.Your ethics statement should only appear in the Methods section of your manuscript. If your ethics statement is written in any section besides the Methods, please move it to the Methods section and delete it from any other section. Please ensure that your ethics statement is included in your manuscript, as the ethics statement entered into the online submission form will not be published alongside your manuscript.

**Reply 4:** Thank you for your guidance. We confirm that the ethics statement has been appropriately included in the Methods section of the manuscript. Additionally, we have removed the duplicate ethics statements from all other sections of the manuscript, as advised. (Page 17, line 483-489 in Revised Manuscript)

5.If the reviewer comments include a recommendation to cite specific previously published works, please review and evaluate these publications to determine whether they are relevant and should be cited. There is no requirement to cite these works unless the editor has indicated otherwise.
**Reply 5:** Thank you for the reminder. We have carefully reviewed all reviewer comments and confirm that neither reviewer recommended citation of any specific previously published works. We have also re-examined our reference list and believe that the cited literature is appropriate, up-to-date, and accurately reflects the background and context of our study. We sincerely appreciate your guidance.

6. Please review your reference list to ensure that it is complete and correct. If you have cited papers that have been retracted, please include the rationale for doing so in the manuscript text, or remove these references and replace them with relevant current references. Any changes to the reference list should be mentioned in the rebuttal letter that accompanies your revised manuscript. If you need to cite a retracted article, indicate the article’s retracted status in the References list and also include a citation and full reference for the retraction notice.

**Reply 6:** Thank you for the reminder. We have carefully reviewed all references cited in the manuscript. To the best of our knowledge, none of the referenced articles have been retracted. Therefore, no changes to the reference list were necessary.

Response to Editor – Regarding Equation Formatting

Thank you very much for your kind reminder regarding the formatting of mathematical expressions. The equation for calculating the age-standardized rate (ASR) was inserted as an image in the original manuscript to ensure clarity, precise alignment, and consistent formatting across platforms, especially considering the complexity of subscripts and summation symbols. We understand that inserting equations using Equation Tools or MathType is generally preferred. However, to maintain visual clarity and layout consistency, we respectfully request to retain the current image-based format of the ASR formula, unless this is strictly against the journal’s production requirements. Please let us know if reformatting remains necessary — we will be happy to comply.

Reviewer #1: This manuscript addresses a relevant subject such as gout and a prevalent condition such as obesity. On behalf of the metodological approach, it is consistent and have been adequately explained. Even though the gap of years from the original data and the analysis exceeds 3 years, the forecast ARIMA is an appropriate complement.

**Reply 1:** We thank the reviewer for the valuable observation. Indeed, our analysis is based on data available up to 2021, and we acknowledge the temporal gap relative to the current year. However, due to the official release schedule of the GBD dataset, 2021 remains the most recent publicly available data at the time of analysis. To address this limitation, we employed the ARIMA model to project trends beyond 2021, providing a forward-looking perspective and enhancing the practical relevance of our findings.

Reviewer #2: This manuscript provides a comprehensive and technically sound assessment of the burden, risk factors, and projected trends of gout in BRICS countries using data from the Global Burden of Disease Study 2021. The research question is clearly articulated, and the use of standardized GBD methodology, joinpoint regression, and ARIMA modeling supports the validity of the findings. The authors succeed in contextualizing gout trends within broader epidemiological and demographic transitions in these emerging economies.
The manuscript makes a valuable contribution to global public health by identifying national disparities, age-specific trends, and sex differences in gout burden. The findings are well supported by data and presented with clarity. The projections until 2036 offer actionable insights for policymakers and healthcare planners. Overall, this is a well-executed and timely study that merits publication after minor revisions. I commend the authors for their rigorous work and the clarity of their presentation.
That said, I offer the following suggestions to further improve the manuscript:
- ①Clarify interpretation of ARIMA projections: The discussion of future trends could benefit from a more cautious tone regarding the limitations of ARIMA models, particularly given the potential for policy or healthcare system changes in BRICS nations over the forecast horizon.

**Reply 1:** We appreciate this insightful suggestion. We have revised the Discussion section to adopt a more cautious tone and elaborated on the potential limitations of ARIMA forecasting, particularly its assumption of historical trend continuation without accounting for future policy shifts, healthcare innovations, or socioeconomic disruptions. (Page 13, line 368-371 in Revised Manuscript)
- ②Language refinement: While the English is generally clear, the manuscript would benefit from professional proofreading to enhance readability and eliminate minor errors or overly long sentences.

**Reply 2:** Thank you for the suggestion. We have carefully revised the language throughout the manuscript and kept all changes visible using Track Changes to demonstrate our thorough editing efforts. We hope the revised version meets your expectations in terms of clarity and readability.

- ③Data visualization: Figures 2 through 5 present complex age and sex-specific trends. Consider simplifying the layout or improving contrast and labels for better readability.

**Reply 3:** Thank you for your valuable suggestion regarding the data visualization in Figures 2 through 5. We carefully re-evaluated these figures and acknowledge the complexity involved in presenting age- and sex-specific trends across multiple countries. However, we believe that the current layout—although detailed—is necessary to accurately convey the nuanced patterns across different age groups, sexes, and BRICS countries. The visualizations were optimized with clear legends, color contrasts, and labels to facilitate interpretation. Given the multidimensional nature of the data, we respectfully propose to retain the existing figure design to preserve the integrity and clarity of the findings. We remain open to further suggestions should the editorial team recommend specific adjustments.

-④Policy implications: The discussion might be strengthened by outlining concrete, country-specific policy recommendations, especially for nations projected to experience an increasing burden (e.g., India, South Africa).

**Reply 4:** We have added a new paragraph in the discussion section highlighting tailored policy recommendations for BRICS countries, with particular emphasis on India and South Africa, where rising trends were noted. These include enhanced screening programs, weight management initiatives, and public health awareness campaigns. Page 14, line 380-388 in Revised Manuscript)
- ⑤Limitations section: The authors do well in acknowledging the limitations of the GBD data and modeling assumptions. Consider expanding this section slightly to highlight the absence of some known gout risk factors (e.g., diet, alcohol use) in the current GBD framework.

**Reply 5:** Thank you for this helpful comment. We have expanded the **Limitations** section to emphasize that while high BMI and kidney dysfunction are included in the GBD framework, other known risk factors such as high-purine diet, alcohol use, or specific genetic predispositions are not currently modeled, which may lead to an underestimation of burden attribution. Page 15, line 418-424 in Revised Manuscript)

We hope that the revisions adequately address all concerns and improve the clarity and quality of our manuscript. We thank the editorial team and reviewers once again for their helpful feedback and guidance.

Yours sincerely,

Jiwen Cheng

[chengjiwen@stu.gxmu.edu.cn](mailto:chengjiwen@stu.gxmu.edu.cn)
