# Supplementary material for: Burden, risk factors, and forecasts of gout in BRICS countries, 1990–2021: Insights from the Global Burden of Disease Study 2021
Source: PLoS One. 2025 Sep 24;20(9):e0332265. doi: 10.1371/journal.pone.0332265 (PMC12459839; doi:10.1371/journal.pone.0332265)
Supplement: Attachment — Submitted filename: Response to the Editor and Reviewers (R2).docx [file pone.0332265.s003.docx]

**Response to the Editor and Reviewers**

Dear Dr. Yang and Reviewers,

We greatly appreciate the chance to resubmit our revised manuscript entitled “Burden, risk factors, and forecasts of gout in BRICS countries, 1990–2021: insights from the Global Burden of Disease Study 2021” (Manuscript ID: PONE-D-25-27682). We are thankful for your guidance throughout the review process and for the insightful comments provided by the reviewers. Based on the feedback, we have made further revisions to improve the clarity and rigor of the work. Our detailed responses to each comment are outlined below, with all changes marked in the manuscript for ease of review.

Reviewer #2: I thank the authors for their detailed and thoughtful responses to the previous round of comments. The revised manuscript has significantly improved in clarity, structure, and depth of discussion. All my prior concerns were adequately addressed, including the interpretation of ARIMA projections, refinement of language, and the inclusion of country-specific policy implications and limitations related to GBD modeling.

Regarding the data visualizations (Figures 2–5), I acknowledge the authors' rationale for maintaining the detailed format, given the multidimensional nature of the data. However, I would like to note that in the current PDF version, the figures appear slightly blurry, making the smaller labels difficult to read. This may be an issue related to PDF rendering or resolution, and I trust it can be resolved during the production and typesetting process.

I commend the authors on this valuable contribution to the literature on gout epidemiology and support the manuscript’s publication.

**Reply:** We sincerely thank the reviewer for the positive and encouraging feedback on our revision, and for acknowledging the improvements in clarity, structure, and discussion. We also appreciate the reviewer’s supportive recommendation for publication. Regarding the minor point about figure resolution in the PDF version, we agree that this is likely related to PDF rendering. We will work closely with the editorial office and production team to ensure that the final published figures are of high resolution and easily legible. We are grateful for the reviewer’s constructive input throughout the review process, which has greatly strengthened our manuscript.

Reviewer #3: The authors conducted a comprehensive and systematic assessment of the burden and projected trends of gout in BRICS countries, utilizing data from the GBD 2021 study. This study performs well in terms of clinical significance and research design, offering a detailed and insightful analysis of gout epidemiology within these nations. Overall, this study delivers valuable evidence on the current burden and future projections of gout in BRICS countries, and provides insights to inform gout prevention and management strategies in BRICS countries. I have some suggestions for this work.

**Reply:** We sincerely thank the reviewer for the positive and thoughtful comments regarding the clinical significance, research design, and overall contribution of our study. We are pleased that the reviewer recognizes the value of our analysis in providing evidence on the current burden and projected trends of gout in BRICS countries, as well as its implications for prevention and management strategies.

We also greatly appreciate the reviewer’s constructive suggestions. We have carefully considered each point and revised the manuscript accordingly to further strengthen the clarity, rigor, and impact of our work. Detailed responses to the specific comments are provided below.
1. Line 124-131: It is not clear how to recognize the gout related burden attributable to high BMI and kidney dysfunction in this study. In other words, how the authors confirm that kidney dysfunction in this study is risk factor of gout? As we all know, there is a bidirectional association between kidney damage and gout.

**Reply 1:** We thank the reviewer for this insightful comment regarding the attribution of gout burden to kidney dysfunction. We fully agree that there is a bidirectional association between gout and kidney damage in clinical practice. However, in the GBD framework, the risk–outcome pairs are predefined based on systematic reviews and evidence of causality. For gout, only two level-2 risk factors are available in the GBD results tool—high BMI and kidney dysfunction. Importantly, in the GBD dataset, kidney dysfunction is modeled as a risk factor contributing to the burden of gout, but not as a downstream outcome of gout. Therefore, our analysis was limited to the directionality defined by the GBD study (i.e., kidney dysfunction→gout), and we could not assess the reverse association (gout→kidney dysfunction), which is not included in the current GBD risk-outcome framework. We have clarified this point in the revised Methods section to avoid ambiguity. (Page 5, line 124-128)
2. Line 184-197: In Figure 2, in addition to the peak age group, the primary affected age range also offers significant insights across countries. Please provide relevant descriptions and discussions to elaborate on these findings.

**Reply 2:** We thank the reviewer for this helpful suggestion. We agree that in addition to the peak age group, describing the broader affected age ranges provides further insights into the epidemiology of gout across BRICS countries. In the revised manuscript, we have added descriptions highlighting the primary age ranges with a high burden of gout in each country, as well as cross-country similarities and differences. We also added a brief discussion on the implications of these age distribution patterns for disease prevention and management. The relevant changes have been incorporated into the Results (Page 7, lines 195–198) and Discussion (Page 10, lines 259–263).

We hope that the revisions adequately address all concerns and improve the clarity and quality of our manuscript. We thank the editorial team and reviewers once again for their helpful feedback and guidance.

Yours sincerely,

Jiwen Cheng

[chengjiwen@stu.gxmu.edu.cn](mailto:chengjiwen@stu.gxmu.edu.cn)
